# Bacterial syntenies: an exact approach with gene quorum

**DOI:** 10.1186/1471-2105-12-193

**Published:** 2011-05-24

**Authors:** Yves-Pol Deniélou, Marie-France Sagot, Frédéric Boyer, Alain Viari

**Affiliations:** 1 INRIA Grenoble-Rhône-Alpes, team BAMBOO, 655 avenue de l'Europe,38334 Montbonnot Cedex, France; 2 Université de Lyon, F-69000, Lyon; Université Lyon 1; CNRS, UMR5558, France; 3 UMR754 INRA, Université de Lyon 1, 50 Avenue Tony Garnier, 69007 Lyon, France

## Abstract

**Background:**

The automatic identification of syntenies across multiple species is a key step in comparative genomics that helps biologists shed light both on evolutionary and functional problems.

**Results:**

In this paper, we present a versatile tool to extract all syntenies from multiple bacterial species based on a clear-cut and very flexible definition of the synteny blocks that allows for gene quorum, partial gene correspondence, gaps, and a partial or total conservation of the gene order.

**Conclusions:**

We apply this tool to two different kinds of studies. The first one is a search for functional gene associations. In this context, we compare our tool to a widely used heuristic - I-ADHORE - and show that at least up to ten genomes, the problem remains tractable with our exact definition and algorithm. The second application is linked to evolutionary studies: we verify in a multiple alignment setting that pairs of orthologs in synteny are more conserved than pairs outside, thus extending a previous pairwise study. We then show that this observation is in fact a function of the size of the synteny: the larger the block of synteny is, the more conserved the genes are.

## Background

The increasing number of fully sequenced microbial genomes (more than 1200 genomes are now completed and available) is fuelling our knowledge on the architecture and dynamics of these bacterial genomes [1]. In this context, the identification of conserved genomic regions across several species is of prime importance. These conserved blocks of genes are referred to in the literature as (micro-)syntenies, (conserved) synteny blocks, (conserved) gene clusters, or gene teams. From an evolutionary stand-point, they are important for understanding how, as species diverge, their gene order is progressively shuffled [2,3]. From a more practical genome annotation perspective, it is also important to identify which regions are resisting this continuous shuffling because these regions are likely to be subjected to stronger functional constraints. In prokaryotic genomes these constraints are of course related to the operon structure. Operons, although subjected as well to gene shuffling [4] appear to be more robust, especially in the case of physically interacting gene products [5].

In recent years, various computational methods have been proposed to identify syntenies by comparison of two or more genomes. In the following, we shall focus on methods working at the gene level, i.e. excluding approaches based on other genetic markers. Even with this restriction, the approaches vary greatly from one study to the other. However, they could be roughly classified into several categories according to their ultimate goal, the algorithmic technique used to solve the problem and the type of constraints they can handle. Among these constraints, the most discriminant ones are i) whether the gene-to-gene (homology) relationship is one-to-one, many-to-many or is an equivalence relation; ii) whether the model accounts for inversions or iii) insertion/deletion events (duplication events are already covered by criterion i), iv) whether the approach is pairwise or is applicable to multiple genomes. Table 1 summarises some important cases found in the literature that can be classified in three main categories.

**Table 1:** Whole genomes alignement methods in the literature

			edition operations				
**Name**	**nb genomes**	**correspondence**	**INV/TRANS**	**DUPL**	**DEL**	**algorithm**	**reference**

GRIMM	pairwise	one-to-one	yes	yes	yes	minimise evolutionary distance	[6]

CINTENY	pairwise	many-to-many	yes	yes	yes	minimise evolutionary distance	[7]

UNOAND TAGIURA	pairwise	one-to-one	yes	no	no	find common intervals	[8]

HEBERAND STOYE	multiple	one-to-one	yes	no	no	find common intervals	[9]

DIDIER	pairwise	many-to-many	yes	yes	no	find common intervals	[10]

GENE TEAMS	multiple	equivalence	yes	no	yes	divide and conquer	[11]

HOMOLOGY TEAMS	pairwise	equivalence	yes	yes	yes	divide and conquer	[12]

DOMAIN TEAMS	multiple	equivalence	yes	yes	yes	divide and conquer	[13]

MCGS	multiple	equivalence	yes	yes	yes	divide and conquer	[14]

MCPAGE	pairwise	equivalence	yes	yes	yes	divide and conquer	[15]

MCMUSEC	multiple	equivalence	yes	yes	yes	divide and conquer	[16]

C3PART	multiple	many-to-many	yes	yes	yes	partition the *NAM*	[17]

FISH	pairwise	many-to-many	local	no	yes	dynamic programming	[18]

DAGCHAINER	pairwise	many-to-many	no	no	yes	dynamic programming	[19]

COLINEARSCAN	pairwise	many-to-many	no	no	yes	dynamic programming	[20]

SYNTENATOR	multiple	many-to-many	no	yes	yes	dynamic programming on POG	[21]

CYNTENATOR	multiple	many-to-many	no	yes	yes	same + phylogeny	[22]

ADHORE	pairwise	many-to-many	no	tandem	yes	clustering	[30]

I-ADHORE	multiple	many-to-many	no	tandem	yes	greedy heuristic	[26]

Principal approaches for whole genomes alignment and the search for syntenies. Note that the real goal of GRIMM and CINTENY is to reconstruct evolutionary scenarios, the extraction of syntenies is only a by-product of those algorithms. Note also that DOMAIN TEAMS uses a protein domain granularity, whereas all other methods operate at the gene level. Among the multiple comparison tools, MCGS (and its extension MCMUSEC), SYNTENATOR (and its extension CYNTENATOR) and I-ADHORE can handle (directly or indirectly) a gene quorum.

The first category contains methods that aim at reconstructing evolutionary scenarios using sorting by reversal to recover the minimal number of permutations needed to transform one genome (modelled as a signed or unsigned permutation of integers) into the other. In such approaches, of which the two main ones are GRIMM [6] and CINTENY[7], the identification of blocks of syntenies is, in some way, a by-product of the algorithm, not its main goal. Some authors also formalise the problem of genome alignment as an exact search for common intervals between permutations. The first article using this formalism was Uno et al. [8]. Two important extensions were proposed later on, an extension to multiple alignment by Hebert and Stoye [9] and another extension to a many-to-many relation by Didier et al. [10].

A second, different, formalism used by several authors is well illustrated by the concept of GENETEAMS[11], proposed originally by Bergeron et al. This defines syntenies as sets of genes such that on each genome no pair of genes from the team are separated by more than *delta *genes, the main limitation being that, in the original formulation, the homology relation is supposed to be one-to-one.

This idea was developed in several papers, He and Goldwasser [12] proposed the notion of HOMOLOGYTEAMS, which allows duplications on a genome; Pasek et al. [13] chose to cut the genes into protein domains to force an equivalence relation. Some other extensions of the model were suggested by Kim et al. (relaxing the proximity constraint on several genomes [14]), and Ling et al. (allowing for overlapping units, for example sequence anchors: [15,16]). More generally these approaches consider the question of finding syntenies as a problem of combinatorial optimisation and often rely on graph theory both to formalise and to solve it. The main difficulty is to limit the combinatorial explosion when the gene-to-gene relationship is not one-to-one. The common components approach proposed by Boyer et al. [17] in a more general biological context belongs to the same family. The approach developed in this paper is an extension of this latter method and will therefore be detailed later on.

In a third category of methods, the problem of genome alignment is treated in a similar way as what is done more classically for pairwise or multiple sequence alignments and relies on a dynamic programming approach to solve it. Three typical examples of such an approach are FISH [18], DAGCHAINER[19] and COLINEARSCAN[20]. The two strong advantages brought by these approaches are i) they do not require an explicit gene-to-gene relationship and can cope with an arbitrary similarity (i.e. substitution) score between genes; ii) as score-based techniques they are more amenable to a statistical evaluation of the significance of the predicted regions. On the other hand, the most important difficulty is to handle inversions in the dynamic programming framework.

Of course, this classification is not perfect and several hybrid methods have already been proposed that belong to several classes. For instance, SYNTENATOR[21] (and its most recent version CYNTENATOR[22]) uses both dynamic programming and a partial order graph representation to detect conserved gene orders in multiple genomes.

In this paper, we introduce an extension of the general graph alignment algorithm presented in [17] and [23] successively. In [17] we introduced the idea of a merged representation of a graph alignment as a multigraph (termed Network Alignment Multigraph (*NAM*)). This corresponds to an idea that was previously more informally stated in [24] and was also developed in [25]. The definition of blocks of synteny (allowing for gene gaps and permutations) then follows from simple properties of this multigraph. This first approach however suffered from two limitations: i) it required the explicit construction of the *NAM*, therefore facing the problem of combinatorial explosion in case of multiple genomes or of a very degenerated gene-to-gene relationship and ii) it assumed that genes are in correspondence when they form a clique (i.e. are all pairwise related). In [23] we proposed, in the context of protein-protein-interaction (*PPI*) networks, to address the first issue by a more careful exploration of the search space, using a depth-first search and "on-the-fly" construction of the *NAM*. We also introduced alternative ways of grouping genes such as stars or, simply, as connected components instead of as complete cliques. In this paper, we increase the flexibility even more by introducing a quorum i.e., when dealing with multiple (*n >*2) genomes, we do not require genes to be present on all but only on at least *q *(≤ *n*) of them. Notice that the algorithm presented in [23] is not restricted to genomes but applies to any kind of graphs. Our extension will apply as well in the general case. In this paper, we shall therefore keep all definitions as general as possible but restrict the illustrations and interpretations to the case of linear graphs representing genomes.

This paper is organised as follows. First, we describe the approach, starting with an informal presentation before going through the definitions, stating precisely what objects we are going to look for, and then what algorithm we are going to use. In the next section, we describe our results, a comparison to an existing method called I-ADHORE[26], and an illustration of the interest of the approach for studies on bacterial evolution.

## Description of the approach

### Informal presentation of the approach

In this section, we first give a brief summary of the approach without quorum, then explain informally how to introduce it.

Given *n *chromosomes represented as interval graphs (i.e. vertices are genes and two genes are connected when they are adjacent on the chromosome, or, more generally, when there are less than *δ_gap _*intervening genes between them), the first step is to define a pairwise correspondence relation (noted *S*) between genes from different chromosomes. Ideally *S *could be homology (i.e. having a common ancestor) or isofunctionality (i.e. having the same function). In practice, both are traditionally approximated by sequence similarity, for instance by thresholding a BLASTP score or using more sophisticated orthologs detection techniques [27,28]. Note that, by contrast to homology and isofunctionality that are both transitive, thresholded sequence similarity and orthology are not necessarily transitive relations.

With this pairwise gene-to-gene correspondence at hand, the next step is to extend it to a multiple (n-way) correspondence. This is done by specifying a topology constraint on *S*. The strongest constraint is that genes connected by *S *form a clique [17] and the loosest constraint is that they merely form a connected component. Intermediate constraints, such as quasi-cliques are also possible [23]. Whatever the choice of this constraint, we end up with *n*-tuples of genes representing the n-way gene correspondence between the *n *genomes.

**Figure 1:**
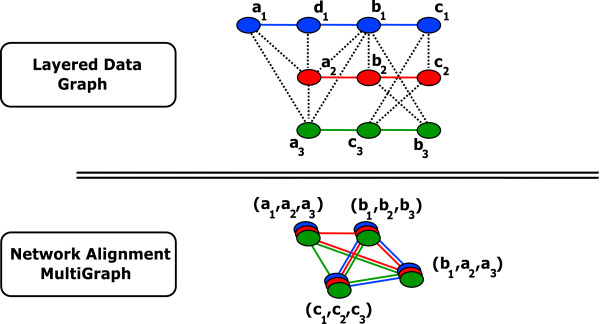
**Example of Network Alignment Multigraph**. A simple example of layered data graph (top) and network alignment multigraph *NAM *(bottom). The layered data graph represents three genomes (blue, red and green). Vertices represent genes and coloured edges represent strict gene adjacency along each genome (no gaps edges in this example). The inter-genomic gene-to-gene correspondence relation *S *is represented by black dotted edges (notice that *S *is neither one-to-one nor transitive). If we choose to associate genes that form cliques of *S *(other choices are possible, see text), then the corresponding network alignment multigraph (*NAM*) is displayed on the bottom. The vertices of the *NAM *are 3 - *uples *(cliques) of genes, also called spines. The coloured edges between spines correspond to the original edges in the layered data graph. For instance, (*a*_1_, *a*_2_, *a*_3_) is red-connected to (*b*_1_, *b*_2_, *b*_3_) because *a*_2 _is connected to *b*_2 _in the red layer of the layered data graph. The same is true for (*b*_1_, *b*_2_, *b*_3_) and (*c*_1_, *c*_2_, *c*_3_) since *b*_2 _and *c*_2 _are connected in the red layer. Conversely, (*a*_1_, *a*_2_, *a*_3_) is not blue-connected to (*b*_1_, *b*_2_, *b*_3_) since there is one gap gene (*d*_1_) on the blue genome separating *a*_1 _from *b*_1 _(see text on how to introduce gaps). Syntons are the sets of spines that are connected for all colours. They form a partition of the *PNAM *vertices. In this case there are 2 syntons: {(*a*_1_, *a*_2_, *a*_3_)} and {(*b*_1_, *a*_2_, *a*_3_), (*b*_1_, *b*_2_, *b*_3_), (*c*_1_, *c*_2_, *c*_3_)}.

These *n*-tuples - also called "spines" [25] - constitute the vertices of a graph representation called Network Alignment Multigraph (*NAM*). These vertices are connected by *n *sets of edges - also called "colours" - corresponding each to the connectivity in a primary interval graph. Figure 1 gives an example of a *NAM *for three genomes where the spines are defined as cliques of the correspondence relation *S*.

In this *NAM*, we then simply define blocks of syntenies -called syntons- as the maximal subgraphs that are connected on each of the *n *colours.

This definition of syntenies matches the intuition that they are made of corresponding neighbourhoods: the selection of spines ensures the gene-to-gene correspondence, and the connectivity condition on each colour ensures that on every genome a synton involves connected genes. Also notice that this definition allows for any permutation in the gene order along the chromosomes.

An important property that follows from this definition is that syntons form a partition of the vertices of the *NAM*. This means that efficient partitioning algorithms can be put into play, provided that the *NAM *has been already built.

But therein lies the rub: in the general case, the *NAM *itself may grow exponentially with the number of genomes, both in terms of vertices and of edges.

In Deniélou et al. [23] we proposed to avoid the explicit construction of the *NAM *by building "on the fly" only the parts of the multigraph we need. The idea is to add the genomes progressively in a depth-first search (and construction) of the multigraph. To do this, we basically alternate two procedures: Split and Expand. Split partitions the current multigraph on the previously treated genomes and Expands adds a new genome.

In this paper, we show that the same kind of strategy also works when allowing for missing genes on several genomes.

The basic idea is that instead of *n*-tuples we now allow for *k*-tuples, with 2 ≤ *q *≤ *k *≤ *n*, where *q *is a quorum fixed by the user. With these *k*-tuples, we can define a Partial Network Alignment Multigraph (*PNAM*) in a similar way as we defined the *NAM *before, and syntons are maximal subsets of *k*-tuples that are connected on the *k *colours.

Intuitively, this means that a synton concerns exactly *k *genomes (with *q *≤ *k *≤ *n*). On these genomes, a synton is made of corresponding neighbourhoods as with the previous definition and the *n - k *remaining genomes are simply ignored.

The next sections explain the formalisation and algorithm in more detail.

### Layered data graph

The layered data graph (also called layered alignment graph in [25]) provides the simplest representation of the primary data at hand.

***Definition***. (adapted from [25]) *Given a set of n primary graphs G_i _*= (*V_i_, E_i_*), *i *∈ [|1, *n|*] *and a correspondence relation S between the elements of distinct sets V_i_, the *layered data graph *is the graph D *= (*V, E*) *with*

• 

• 

Observe that there are two kinds of edges in *E*: edges in *E_P _*correspond to the original sets *E_i _*(here-after called *intra-layer edges*) and the other ones (*E_S_*) connect vertices from different layers (here-after called *inter-layer edges*) (see Figure 1).

In the specific case of genomes, *V_i _*represents the set of genes in the *i^th ^*genome and *E_i _*represents gene contiguity: (*u_i_, v_i_*) ∈ *E_i _*⇔ *|rank*(*u_i_*) - *rank*(*v_i_*)| ≤ *δ_gap _*where *rank *is the rank of the gene on the chromosome and *δ_gap _*is a gap parameter (the formula can easily be adapted to circular genomes as well).

### *n*-way partial correspondence and don't care element

As mentioned earlier, dealing with *n *≥ 2 genomes requires to define formerly how genes from different genomes are aggregated.

The definition of an *n*-correspondence without a quorum (i.e. for *q *= *n*) is the following:

***Definition (without quorum)***. *An n*-way correspondence *between elements of V*_1_, *V*_2_,..., *V*_*n *_*is defined as a restriction **of the cartesian product, denoted by *.

Several practical cases of such an aggregation are discussed in [23].

For instance, a clique aggregation requires that all elements of an *n*-tuple are pairwise related: , *v*_i _*S v*_*j*_.

Conceptually the introduction of a quorum *q *consists of working with *k*-tuples of genes (with *q ≤ k ≤ n*) instead of *n*-tuples. However for the sake of simplicity, we shall continue to work with *n*-tuples by introducing a *don't care *element, *.

Given , we introduce a function called *cover *which for a given *n*-tuple *v *returns the set of nodes in *v *that are not *don't care *elements. Formally, , *cover*(*v*) = {*v_i _*≠ _*_, *i *∈ [|1, *n|*]}.

One can now define an *n*-way partial correspondence.

***Definition (with quorum)***. *An n*-way partial correspondence *between elements of V*_1_, *V*_2_,..., *V*_*n *_*for a quorum q is defined as an n-way correspondence **such that *, |*cover*(*v*)| ≥ *q*.

*We shall in the following use the notation *.

As before, this restriction is computed by using the *S *relation. For instance a clique aggregation for a quorum *q *would be expressed as follows  and ∀*i, j*, (*v*_*i *_*S v*_*j*_) or (*v*_*i *_= _*_) or (*v*_*j *_= _*_).

### Partial Network Alignment MultiGraph

The *PNAM *(Partial Network Alignment Multigraph) is an extension of the *NAM *(Network Alignment Multigraph) presented in [23]. It summarises both the *n*-way partial correspondence and the connectivity in the genomes (i.e. gene neighbourhood).

***Definition (with quorum)***. *A *partial network alignment multigraph (*PNAM*) *for n primary graphs G*_*i *_= (*V*_*i*_, *E*_*i*_) *is a graph **such that*:

• 

• *and *, 

In other words, the vertices of the multigraph are *n*-tuples of genes and *don't care *elements and there is an edge *e *∈ ℰ*_i _*(that is, an edge of colour *i*) between two vertices if they have genes at the *i^th ^*position that are neighbours in the genome *G_i_*. In the following, we refer to such an edge as an *edge of colour i*.

**Figure 2:**
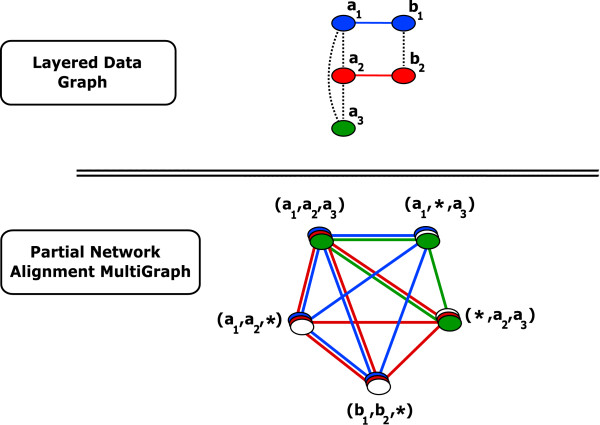
**Example of Partial Network Alignment Multigraph**. A simple example of layered data graph (top) and partial network alignment multigraph *PNAM *(bottom). As in Figure 1, the *S *gene-to-gene relation is represented by dotted edges. Vertices of the *PNAM *(spines) correspond to cliques of the *S *relation. The difference with a *NAM *(Figure 1) is that "*don't care*" genes (represented as _*_) are now allowed in spines (here we have a quorum *q *= 2 which means we cannot have more than one _* _in a spine). The set of vertices of this *PNAM *can be partitioned into four syntons, three of them are singletons: {(*a*_1_, *a*_2_, *a*_3_)}, {(*a*_1_, _*_, *a*_3_)} and {(_*_, *a*_2_, *a*_3_)}, the fourth is of size 2: {(*a*_1_, *a*_2_, _*_), (*b*_1_, *b*_2_, _*_)}. Only two of them ({(*a*_1_, *a*_2_, *a*_3_)} and {(*a*_1_, *a*_2_, _*_), (*b*_1_, *b*_2_, _*_)}) are maximal.

Figure 2 gives an example of layered data graph for three genomes and the corresponding *PNAM *for a clique aggregator with *q *= 2.

### Defining syntons in the *PNAM*

With the previous definition of a *PNAM *at hand, several definitions of synteny are possible, depending on the properties one is looking for (see Table 1).

For instance, without quorum (i.e. *q *= *n*) and if strict gene order has to be conserved, syntons are simply the connected components of the *PNAM *in which all edges that do not have all of the *n *colours are removed. Note that it is also possible to specify a partial gene order conservation, stating that, in a given synton, two spines consecutive for a colour should not be separated by more than *δ_shuffle _*spines for the other colours. This idea will not be developed in the present paper.

Since we want to allow all gene permutations, we shall use the most general definition, which is the following.

***Definition (without quorum)***. *A *synton *is a maximal subgraph **of the PNAM such that *∀*i *∈ [|1, *n*|], *is connected for *.

To introduce a quorum, one has to modify the previous definition in order to cope with the presence of *don't care *elements in the *PNAM *vertices.

***Definition (with quorum)***. *A *synton *is a maximal subgraph **of the PNAM such that *∃*I *⊆ [|1, *n*|], |I| ≥ q such that ∀*i *∈ *I*, *is connected for **and *, *cover*(*v*) = *I*.

Informally, the first part of the definition ensures that the result will be a synton when restricted to the colours *I*, and the second part makes sure that for the colours *i *∉ *I*, all the vertices of  correspond to a *don't care *element. In the previous definition, the term "maximal" naturally means that no other vertex of the *PNAM *can be added without breaking the connectivity conditions. With this definition, it is easy to show that syntons form a partition of the *PNAM *vertices.

The *PNAM *given in Figure 2 is thus partitioned into 4 syntons: {(*a*_1_, *a*_2_, *a*_3_)} (*I *= {1, 2, 3}), {(*a*_1_, _*_, *a*_3_)} (*I *= {1, 3}), {(_*_, *a*_2_, *a*_3_)} (*I *= {2, 3}) and {(*a*_1_, *a*_2_, _*_), (*b*_1_, *b*_2_, _*_)} (*I *= {1, 2}).

However, one can notice that although each class of this partition is maximal in the previous sense, it may not be maximal in terms of the genes involved. When projecting a synton onto the layered data graph, it may occur that the set of genes obtained is included into another projection (this occurs for instance when a synton on *k *genomes has the same boundaries as a synton on *k *+ 1 genomes). It is therefore advisable to add another constraint to syntons in order to remove some redundancy in the results in terms of genes.

***Definition of ***⊑. *Let us denote by *⊑ *the relation defined as follows: for u*, *u *⊑ *v *⇔ *cover*(*u*) ⊆ *cover*(*v*).

This means that all vertices in the spine *u *that are not "don't care" elements are also elements of the spine *v*.

This definition can be extended to subgraphs of the *PNAM*.

***Definition of ***⊑. *For two subgraphs of the PNAM C*_1 _= (*U*_1_, *F*_1_,... *F*_*n*_) *and *, *C*_1 _⊑ *C*_2 _⇔ ∀*u*_1 _∈ *U*_1_, ∃*u*_2 _∈ *U*_2_, *such that u*_1 _⊑ *u*_2_.

We call then a *maximal synton *a synton that is maximal for the relation ⊑.

On the example of Figure 2, we have for instance {(*a*_1_, _*_, *a*_3_)} ⊑ {(*a*_1_, *a*_2_, *a*_3_)} which means {(*a*_1_, _*_, *a*_3_)} is not maximal. The only two maximal syntons in that example are {(*a*_1_, *a*_2_, *a*_3_)} and {(*a*_1_, *a*_2_, _* _), (*b*_1_, *b*_2_, _*_)}.

Note that this constraint is not embedded into the algorithm but is added as a final filter on the results.

### Algorithm

The most natural approach to compute syntons with quorum would be to build the *PNAM*, and then to use one of the graph partitioning algorithms at our disposal [17,29].

However, we already showed in [23] that when the correspondence relation is not one-to-one, the size of the *NAM *may grow exponentially with the number of graphs. The situation for *PNAM *is even worse since for each vertex of the *NAM *we now have  additional vertices in the *PNAM*. This means that avoiding the explicit construction of the *PNAM *is an even bigger priority.

In this paper, we choose to extend the graph partitioning algorithm described in [23]. The idea is to conduct a depth first search (*DFS*) of the classes starting with the connected components of the first colour (genome), and to add colours incrementally. Therefore the multigraph is not computed explicitly but instead smaller parts of it (classes) are computed on the fly in each branch of the *DFS*.

The basic algorithm is fully described in [23] and a summary pseudo-code is given below. The two main operations are:

• *SPLIT*_1...*i *_that splits a class on colours 1 to *i*;

• *EXPAND*_*i*+1 _that adds the (*i *+ 1)^*th *^colour to the current network alignment multigraph.

**Algorithm 1**: OTF.

**Global**: Layered Data Graph *D *= (*V, E*)

for the primary graphs *G*_*i *_= (*V*_*i*_, *E*_*i*_), *i *∈ [|1, *n*|]

**Input**: Multigraph *C*

/* current class: initialised with */

Integer i/* current layer: initialised with 1 */

**Variables**: Partition *P*

/* current partition on the i first layers */

(1)   **begin **/* partition on the i first layers */

(2)      *P *← *SPLIT*_1...*i*_(*C*),

(3)      **if **(|*P*| ≠ 1) **then **/* C is split */

(4)         **for ***NewC *∈ *P ***do**

(5)            OTF(*NewC, i*)

(6)         **end for**

(7)      **else if **(*i *<*n*) **then **/* C is stable */

(8)         *NC *← *EXPAND*_*i*+1_(*C*),

(9)         OTF(*NC, i *+ 1),

(10)      **else **/* C is stable for all colours */

(11)         PRINT(*C*)

(12)      **end if**

(13)   **end**

#### Initialisation

As in [23], the algorithm is initialised with all connected components on the first genome. In order to cope with missing genes on the first genome, one has only to add an initial singleton class containing a *don't care *vertex.

#### Expand

The procedure called *EXPAND_V ERTICES*_*i*+1 _expands the current set *C *of *PNAM *vertices to two kinds of new (*i *+ 1)-tuples.

The first case is similar to the no-quorum condition and expands each vertex *v *= {*v*_1_, *v*_2_,... *v*_*i*_} ∈ *C *by genes *v*_*i*+1 _∈ *V*_*i*+1_. These genes are called terminals of *v*. Ideally, a terminal *v*_*i*+1 _is such that there exists an *n*-tuple  such that ∀*j *∈ [|1, *i *+ 1|], *u*_*j *_= *v*_*j *_(i.e. *v *is a prefix of *u*). In practice, the efficient computation of these terminals greatly depends upon the chosen aggregation function. For instance, for the clique, this is a simple task: (*v*_1_,..., *v*_*i*+1 _) is required to be a clique too (*don't care *elements are ignored) since this is a necessary condition for the final *n*-tuple to be a clique. Using the ordering *G*1, *G*2, *G*3 in the example given in Figure 2, this allows us to build the vertex (*a*1, *a*2, *a*3) from the vertex (*a*1, *a*2).

The second case is to extend *v *= (*v*_1_,... *v*_*i*_) by a *don't care *element if this is allowed by the quorum condition, i.e. the total number of *don't care *elements is less than *n -q*. These added are intended to introduce the missing genes on genome *i *+ 1. For instance, in Figure 2, with the same ordering *G*_1_, *G*_2_, *G*_3_, the vertex (*c*_1_, *c*_2_, _*_) is recovered thanks to the introduction of *don't care *elements as terminals (it is an expansion of the vertex (*c*_1_, *c*_2_)).

#### Split

As in [23], the *SPLIT*_1*-i *_operation computes the connected components on each colour in turn. If, for a colour *j*, the class is split, then it returns the split parts.

The main difference is that the connected components for a colour *j *are now computed on *P_j_*(*V*), the restriction of the set of vertices to those that do not have a *don't care *element at position *j*.

This is simply done by:

1. removing temporarily all tuples having a *don't care *element at position *j*,

2. computing the connected components of the resulting set of vertices,

3. adding back the previously removed set in each connected component.

Finally, just as in [23], when a class *C *is such that *SPLIT*_1*-i*_(*C*) = *C *then it is stable for colours 1 to i and needs expansion to the (*i *+ 1)*^th ^*colour.

## Results and Discussion

In order to illustrate our approach in practice, we performed two different experiments. The first one is a comparison to a popular heuristics with a similar aim (I-ADHORE). The second one is a study of the evolution rate of genes in synteny that generalises previous observations on this subject. In the remaining of this paper, we shall refer to our algorithm as OTFQ (OTF was the name of the algorithm in [23] and *Q *stands for quorum).

### Comparison to I-ADHORE

In this section, we compare the syntenies recovered by our approach to those found by I-ADHORE[26], a popular program used to recover syntenies with missing genes.

### ADHORE*and *I-ADHORE

ADHORE[30] (Automatic Detection of Homologous Regions) looks for genomic regions between two genomes where the gene order is strictly conserved. The gene-to-gene (homology) relation *S *is, as in our approach, boolean and provided as input to the program together with the genes location. The algorithm basically proceeds by clustering of the homologous genes pairs based on the linear distance of the corresponding genes on both chromosomes (two pairs are close if they lie close together on the same diagonal of the alignment matrix) and a measure of the cluster linearity (how the set of pairs fits to a diagonal). The procedure allows for gaps (maximum number of intervening non-homologous genes between two pairs) but not explicitly for genes permutations although a limited amount of permutations is possible by considering them as gaps.

In 2004, the authors extended this method to the case of multiple genomes: the new version is called I-ADHORE[26]. I-ADHORE starts by collecting the results of ADHORE obtained on each pair of genomes. The results are called "multiplicons of level 2". This series of multiplicons initialises a set ℳ that will eventually constitute the solution set.

The procedure starts with the largest (in terms of gene pairs) multiplicon and represents it as a "profile" i.e. a series of aligned positions without permutation. Next, this profile is aligned to the whole set of chromosomes using a variant of ADHORE. One important point is that there is a match between a gene and a given position in the profile when this position contains at least one gene that matches. In other terms, in I-ADHORE, spines are connected components of the *S *relation.

The results of this alignment are multiplicons of level 3 (they can be extensions of part or the totality of the original multiplicon). These new multiplicons are then put back in the ℳ set and the algorithm iterates. There is no need in I-ADHORE for the quorum parameter we previously defined, instead the results are presented in the form of an arborescence of multiplicons (from level 2 to, possibly, level *n*) from which the spines involving at least *q *genomes can be easily retrieved.

Finally, this algorithm was improved in 2007: in this latest version - I-ADHORE 2.0 [31] - a new alignment profile is recomputed for all segments of the current multiplicon after extension. This implies that some erroneous decisions taken at the beginning of the execution could be corrected later on.

Because I-ADHORE is a heuristic (basically a greedy algorithm), its main advantage is that it is extremely quick. The main drawback is that the definition of a multiplicon is procedural rather than formal. This makes the comparison to other approaches more difficult since we do not know exactly what is to be found and what is missed. Because of this, in the following experiment we decided to compare the results in terms of genes involved in syntons (OTFQ) versus multiplicons (I-ADHORE).

#### Data and parameters

In order to compare the two algorithms in various phylogenetic situations, we constituted four groups, each made of 5 or 10 bacterial species at variable phylogenetic distances (Table 2). The most heterogeneous group is the group **bacteria**, made of phylogenetically distant species. The most homogeneous is the group **entero **made of *Enterobacteriaceae*. The precise composition of each bacterial group is given in Table 2.

**Table 2:** The four groups of bacterial species used in this study

bacteria	Bacteria		
*Acnum*	*Species*	*Mb*	*NbGenes*

AE000512_GR	Thermotoga maritima (strain JCM 10099/DSM 3109)	1.9	1853

AE009951_GR	Fusobacterium nucleatum nucleatum (strain JCM 8532)	2.2	2069

AL009126_GR	Bacillus subtilis (strain 168)	4.2	4237

BA000022_GR	Synechocystis sp. (strain PCC 6803)	3.6	3166

U00096_GR	Escherichia coli (strain K12)	4.6	4320

AE000520_GR	Treponema pallidum (strain Nichols)	1.1	1028

AE001273_GR	Chlamydia trachomatis (strain D/UW-3/Cx)	1.0	895

AM398681_GR	Flavobacterium psychrophilum (strain JIP02/86)	2.8	2432

BX248353_GR	Corynebacterium diphtheriae (strain NCTC 13129)	2.5	2317

CP000359_GR	Deinococcus geothermalis (strain DSM 11300)	2.5	2330

**proteo**	**Bacteria; Proteobacteria**		

*acnum*	*Species*	*Mb*	*NbGenes*

AE005673_GR	Caulobacter crescentus (strain CB15/ATCC 19089)	4.0	3738

AE016825_GR	Chromobacterium violaceum (strain IFO 12614)	4.7	4407

AE017282_GR	Methylococcus capsulatus (strain Bath/NCIMB 11132)	3.3	2960

CP000661_GR	Rhodobacter sphaeroides (strain ATCC 17025)	3.2	3111

U00096_GR	Escherichia coli (strain K12)	4.6	4320

CP000112_GR	Desulfovibrio desulfuricans (strain G20)	3.7	3775

AE001439_GR	Helicobacter pylori (strain J99)	1.6	1491

CP000251_GR	Anaeromyxobacter dehalogenans (strain 2CP-C)	5.0	4346

CP000744_GR	Pseudomonas aeruginosa (strain PA7)	6.6	6286

CP000814_GR	Campylobacter jejuni (subsp. jejuni, serovar O:6)	1.6	1626

**gamma**	**Bacteria; Proteobacteria; Gammaproteobacteria**		

*acnum*	*Species*	*Mb*	*NbGenes*

AE017282_GR	Methylococcus capsulatus (strain Bath/NCIMB 11132)	3.3	2960

CP000127_GR	Nitrosococcus oceani (strain ATCC 19707/NCIMB 11848)	3.5	2976

CP000462_GR	Aeromonas hydrophila (subsp. hydrophila, ATCC 7966)	4.7	4122

CP000744_GR	Pseudomonas aeruginosa (strain PA7)	6.6	6286

U00096_GR	Escherichia coli (strain K12)	4.6	4320

CP000675_GR	Legionella pneumophila (strain Corby)	3.6	3204

CP000681_GR	Shewanella putrefaciens (strain CN-32/ATCC BAA-453)	4.7	3972

CP001091_GR	Actinobacillus pleuropneumoniae (serovar 7, AP6/AP76)	2.3	2131

CP001132_GR	Acidithiobacillus ferrooxidans (strain ATCC 53993)	2.9	2826

AM920689_GR	Xanthomonas campestris (pathovar campestris)	5.1	4510

**enterob**	**Bacteria; Proteobacteria; Gammaproteobacteria; Enterobacteriales; Enterobacteriaceae**		

*acnum*	*Species*	*Mb*	*NbGenes*

AE006468_GR	Salmonella typhimurium (strain ATCC 700720)	4.9	4455

AE009952_GR	Yersinia pestis (biovar Mediaevalis, strain KIM5)	4.6	4104

CP000822_GR	Citrobacter koseri (strain ATCC BAA-895)	4.7	5003

CP000964_GR	Klebsiella pneumoniae (strain 342)	5.6	5425

U00096_GR	Escherichia coli (strain K12)	4.6	4320

AE005674_GR	Shigella flexneri (serovar 2a, strain 301)	4.6	4395

AP008232_GR	Sodalis glossinidius (strain morsitans)	4.2	2432

BX470251_GR	Photorhabdus luminescens laumondii (strain TT01)	5.7	4897

BX950851_GR	Erwinia carotovora (subsp. atroseptica, ATCC BAA-672)	5.1	4491

CP000653_GR	Enterobacter sp. (strain 638)	4.5	4115

Detail of the four groups of bacterial species at various phylogenetic distances used in this study. Each group is composed of 5 (first 5 lines) or 10 species. *acnum*: accession number in the *EMBL/Genome Reviews *databank; *Mb*: size of the genome in Mb; *NbGenes*: number of protein-encoding genes in the genome.

The gene-to-gene relation *S *is provided by BLASTP by selecting pairs of gene products with *p - value ≤ *1*e *- 10, %*identity ≥ *40 and with the alignment covering at least 80% of the smallest protein.

OTFQ was run with the following parameters: gaps of at most 3 genes (*deltagap *= 3), all gene permutations allowed (*deltashuf *= *∞*), minimum synton size of 3 (*mineltsize *= 3) and a quorum *q *of at least 2 genomes. Spines are defined simply as connected components (CC) of *S*. Note that OTFQ allows other definitions of spines, as cliques or *γ*-quasi-cliques of *S *(for a spine made of *n *nodes, it means that for each node *v *we must have *degree*(*v*) ≥ γ _* _(*n - *1)), those definitions are not used in this test.

For I-ADHORE, we used the default parameters, except for "gap_size", "tandem_gap_size" et "cluster_gap" which were all set to 3 in order to be as consistent as possible with the *deltagap *parameter used in OTF. The "anchor_points" parameter, corresponding to the minimal number of genes in a cluster, was set to 3 in order to fit with the "mineltsize" parameter of OTF. Additionally, we set up the *q_value _*parameter very close to 0 and the *prob_cutoff _*parameter to 1, in order to disable any filtering and keep as many multiplicons as possible.

Notice that the chosen parameters are not optimal (both for I-ADHORE and OTF) but were selected in order to make the comparison as consistent as possible. In particular, a quorum of 2 out of 10 genomes is usually too low for OTF since it potentially leads to an exponential growth of the solution size. However, we keep this value since I-ADHORE naturally reports these multiplicons too.

#### Execution times

The execution times of I-ADHORE and OTF for the different groups are given in Table 3.

**Table 3:** Comparison of execution times and results of I-ADHORE and OTFQ

		I-ADHORE	OTFQ	
	
Set		execution time (s)		
	
bacteria	5	36	20	
	10	122	55	
	
proteo	5	110	28	
	10	428	197	
	
gamma	5	232	42	
	10	825	373	
	
entero	5	632	273	
	10	2881	*NA*	
		**number of genes found**		**size of *∩***

bacteria	5	460 (3%)^1^	572	457 (99%)^2^
	10	831 (3%)	919	756 (91%)

proteo	5	2012 (11%)	2449	1991 (99%)
	10	4117 (11%)	4935	4088 (99%)

gamma	5	4266 (21%)	4777	4246 (100%)
	10	8005 (22%)	9039	7977 (100%)

entero	5	15376 (66%)	15792	15355 (100%)
	10	29306 (66%)	*NA*	*NA*

Comparison of execution times (top) and results (bottom) of I-ADHORE and OTFQ for the four groups of 5 and 10 species in Table 1.

^1 ^number of genes found/total number of genes

^2 ^size of *∩ */number of genes found by I-ADHORE

We notice that for the more heterogeneous groups (**bacteria, proteo, gamma**), the OTFQ execution times are short, and indeed, shorter than for I-ADHORE. Both are anyway shorter than the time needed to perform the initial all-against-all BLASTP comparisons needed to compute the *S *relation. However, this situation is inverted when working with close species and a larger number of genomes. For 10 enterobacteria, OTFQ runs out of memory (the limit was 2 Gb). Indeed a closer examination of running times shows that I-ADHORE behaves roughly quadratically with the number of genomes (as expected if the computation time is dominated by the initial pairwise comparison and not by the following greedy search) whereas the OTFQ runtime grows exponentially with this number. However, Table 3 shows that for up to 10 genomes, the problem remains amenable to an exact algorithm and there is therefore no need to resort to heuristics. We should observe however that I-ADHORE was specifically designed to deal with large eukaryotic genomes, whereas our definitions and algorithm are better suited to the alignment of bacterial genomes (one of the differences, for instance, is that we do not look for intra-genomic syntenies).

#### Comparison of results

As mentioned before, in order to compare more easily the results of I-ADHORE (multiplicons) and OTFQ (syntons), we choose to project them back onto the layered data graph (i.e. genomes) and, therefore, to compare sets of genes involved in multiplicons versus syntons. In this comparison, "gap" genes (i.e. genes not involved in any spine for OTFQ and non "anchor" genes for I-ADHORE) are ignored. This is shown in Table 3. We first observe that the number of genes found in multiplicons and syntons are very close, they range from 3% of the total number of genes for distant species (**bacteria **group), and to up to 66% for very close species (**entero **group). Moreover, OTFQ constantly finds slightly more genes than I-ADHORE (from 10 to 20% irrespective of the phylogenetic group) and, except for the 10-**bacteria **group where the overlap is 91%, the set of genes found in multiplicons are almost totally included in the sets found in syntons (from 99 to 100%). A closer examination of these results shows that missed genes come from three main reasons, all of them being related to the linearity and collinearity constraint in I-ADHORE. The first, and more important, reason comes from the definition of "gap" genes. In OTFQ "gaps" are simply genes that do not belong to a spine within the considered synton (they may have no homologous genes or they may be involved in a spine from another synton). In I-ADHORE the definition is similar but multiplicons have an additional linearity constraint that may be broken when the number of "gaps" on one genome is not counterbalanced by an equivalent number of "gaps" on the other one. With 3 "gaps" for instance, a synteny can be missed when two genes are adjacent on one chromosome and their homologous genes are separated by more than 2 gaps on the other chromosome. This linearity condition makes the interpretation of the "gaps" parameter more delicate. Unfortunately, we did not find any I-ADHORE parameter to change this behaviour but we believe this could be fixed easily. The second reason is due to the existence of gene order permutations and is therefore more fundamentally related to the I-ADHORE algorithm, that enforces strict collinearity of genes. Finally, a third case marginally arises from an inversion of the orientation of one gene. This comes from the fact that I-ADHORE considers gene orientation in its definition whereas OTFQ ignores it.

**Figure 3:**
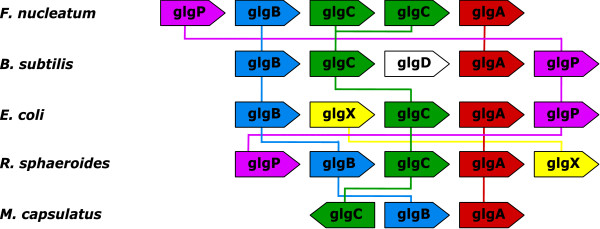
**First Example of Operon with Permutations**. The glycogen biosynthesis/degradation operon in 5 bacterial genomes. Connected components of the *S *relation are represented by coloured lines.

Figure 3 is an illustration of gene order permutation in the case of the glycogen metabolism. Glycogen is the major reserve of polysaccharide in bacteria, its biosynthesis from glucose-1-phosphate is performed in three steps by GlgC (ADP-glucose pyrophosphorylase), GlgA (glycogen synthase) and GlgB (branching enzyme) in that order [32]. Two additional enzymes, GlgX (glycogen debranching enzyme) and GlgP (gycogen phosphorylase), are involved in the reverse process, i.e. in glycogen degradation. All of the corresponding genes are often found clustered together in single or adjacent operons [32,33] but in various orders. The most frequent order for the *glgA/B/C *triplet is BCA (*e.g*. in E. coli on Figure 3) but the order CBA is also observed (*e.g*. in *M. capsulatus *in Figure 3, that also displays an inversion of *glgC*. Finally the order BAC, although rare, is observed too (in *Clostridium perfringens *for instance (data not shown)). The genes *glgP *and *glgX *are not always present close to the *glgA/B/C *triplet but, when they are, their order is very variable. For instance, *glpP *lies before *glgB *in *F. nucleatum *and *R. sphaeroides *but after *glgA *in *B. subtilis *and *E. coli*. The same situation holds for *glgX *in *E. coli *and *R. sphaeroides *(Figure 3). Finally, it is important to notice that the GlgA sequence is not well conserved across bacterial species but is actually split in two groups [32], one is characteristic of *Bacilli*, *Fusobacteria *and *Clostridia*, the second one is characteristic of *Alpha *and *Gamma proteobacteria*. This is the reason why, at a reasonable BLASTP threshold, the GlgA group (red lines in Figure 3) does not form a single connected component but two disjoint components. When these five species are subjected to analysis by the two programs, they give rise to different multiplicons/syntons, reflecting the different ways permutations are handled. I-ADHORE gives rise to 3 multiplicons of level 2 (i.e. involving only pairs of genomes). Each of them is composed of 3 genes: *F. nucleatum/E. coli *{*B, C, P *}, *F. nucleatum/B. subtilis *{*A, C, B*} and *E.coli/R. sphaeroides *{*A, C, B*}. Let us notice that the *E.coli/B. subtilis *{*B, C, P *} is missed because the two gaps in *B. subtilis *break the multiplicon collinearity condition. By contrast, OTFQ finds 4 syntons of larger size. The *E.coli/R. sphaeroides *synton now involves all of the 5 genes: {*A, B, C, P, X*}. Similarly the *F. nucleatum/B. subtilis *now involves 4 genes {*A, B, C, P *}. The *E.coli/R. sphaeroides *synton includes *M. capsulatus *as well: {*A, B, C*}. And finally, OTFQ also finds a synton, {*B, C, P *}, with 4 species: *F. nucleatum/B. subtilis/E. coli/R. sphaeroides*. Notice that, because of the broken connection on *glgA*, no multiplicon nor synton of size at least 3 genes can be found on all of the five species depicted here.

**Figure 4:**
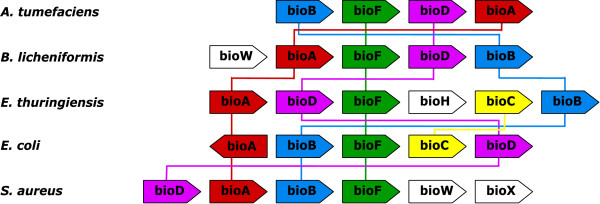
**Second Example of Operon with Permutations**. The biotin biosynthesis operon in 5 bacterial genomes. Connected components of the *S *relation are represented by coloured lines.

Another example of conserved gene neighbourhood with gene permutation is provided by the biotin (vitamin H) biosynthetic pathway. Although biotin is an essential enzyme cofactor in all forms of life, its detailed biosynthetic pathway is not yet fully understood [34]. Indeed, the well documented part of the pathway comprises the four late steps, leading to biotin from pimeloyl-CoA and catalyzed by BioF, BioA, BioD and BioB [35]. In bacteria, the four corresponding genes usually form an operon (or regulon) cluster [35]. This cluster sometimes includes additional *bio *genes involved in the earlier steps leading to pimeloyl-CoA. For instance BioW synthesizes pimeloyl-CoA from pimelic acid and BioC and BioH have recently been suggested to participate in the synthesis of pimeloyl-CoA from malonyl-CoA [34]. Figure 4 displays the gene layout of the *bioF/A/D/B *core cluster in five bacterial species. As shown, each species displays a specific gene order: BFDA (*A*. *tumefaciens*), AFDB (*B. licheniformis*), ADFB (*B*. *thuringiensis*), ABFD (*E. coli*) and DABF (*S. aureus*). Because of this high level of gene shuffling, *i - ADHoRe *was only able to retrieve a single multiplicon involving two species (*A. tumefaciens *- *E*. *coli*) and three genes (*bioB/F/D*). The *bioA/B/F *cluster between *E. coli *and *S. aureus *was missed because of the opposite gene orientation of *bioA*. By contrast, OTFQ could retrieve three different syntons, of much larger sizes. The first synton involves the four *bioF/A/D/B *genes in all species but *S. aureus*. This comes from the fact that *bioD *is not well conserved in *S. aureus *and was therefore not connected to any other *bioD *gene at the selected *BLASTP *threshold. The second synton is therefore composed of the three *bioF/A/B *genes, in all of the five species. Finally, the third synton is composed of the four *bioW/A/F/B *genes in *B. licheniformis *and *S. aureus*. Again, *bioD *is missing from this latter synton because of a lack of sequence similarity. The same is true for *bioC *between *B. thuringiensis *and *E. coli*.

### Synteny and gene evolutionary rates

As mentioned in the introduction, bacterial syntenies can provide useful information about evolutionary processes. For instance, Lemoine et al. have shown [36] that genes in synteny groups are subjected to stronger evolutionary pressure than genes outside of synteny groups. They started by identifying pairs of orthologous genes between two bacterial genomes. A given pair is then classified as a *POG *(Positional Ortholog Group) if it is strictly adjacent to (at least) another pair of orthologous genes. Finally, the *PAM *distances (as computed with the DARWIN package [37]) between the elements of a pair are computed for the *POG *and non-*POG *groups and their distribution are compared.

Using this approach on several pairs of bacterial species, from closely related enterobacteria (*E. coli *and *S. enterica*) to more distant species (*E. coli *and *B. subtilis*), they showed that the mean *d_PAM _*in *POG*s is significantly lower than the mean *d_PAM _*in non-*POG*, thus indicating that genes within synteny groups are generally more conserved than genes outside of synteny groups.

In this section, we attempt to generalise this result in two ways: first by working with more than two genomes, and second by investigating if the same evolutionary trends is observed as a function of the size of the synteny group.

**Figure 5:**
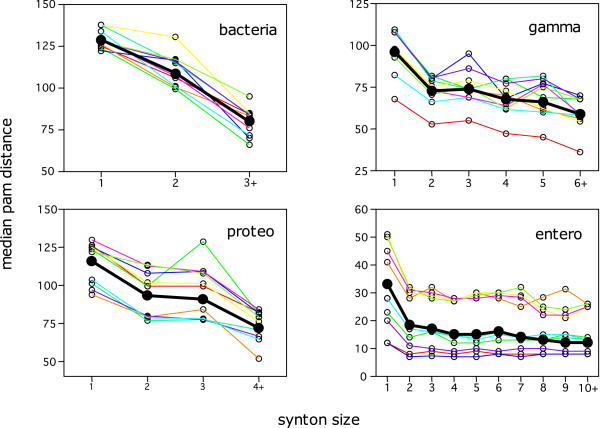
**Distribution of *d*_*PAM*_**. Distribution of *d*_*PAM *_within each group of *max_size _*(size of the maximal synton in which a given pair of orthologs appears). The black curve corresponds to the median of *d_PAM _*on the whole set, whereas the coloured curves correspond to the analysis split by pairs of species. We observe in all cases a clear tendency for *d_PAM _*to decrease when the synteny size increases.

To this purpose, we use the same four sets of five species (**bacteria**, **proteo**, **gamma **and **entero**) as defined before. In the context of an evolutionary study, we need to modify our previous definition of the gene-to-gene correspondence since we want to enforce a true orthology relationship and not simply a sequence similarity relationship. To this purpose, we make use of the INPARANOID program [27]. Two genes from different genomes are related by *S *if they belong to the same group of co-orthologs provided by INPARANOID. Moreover, in order to enforce a more stringent definition of synteny, we use a gap parameter of 0, a quorum of 3 genomes and we force spines to be cliques of the *S *relation. This means that a spine is composed of at least three genes that should be all pairwise related by *S*. We then run OTFQ on the four sets separately (with a minimum synton size of 1, in order to recover isolated spines as well) and we further process the result in the following way. For a given pair of *S *related genes, we record the size of the largest synton to which it belongs (let us denote it by *max_size_*), we compute its *PAM *distance using the same procedure as in [36] (denoted by *d_PAM _*) and we then analyse the distribution of *d_PAM _*within each group of *max_size_*. The results are presented, for each of the four species group, in Figure 5 where the black curve displays the median of *d_PAM _*as a function of the synteny size (*max_size_*). In order to get sufficient statistics (we requested a minimum of 500 pairs in each bin), values of *max_size _*greater than a certain limit are pooled within the same bin (indicated by the "+" postfix in the figure). A first point to observe is the value of the median for *max_size _*= 1 (i.e. for pairs of orthologs only present in isolated spines) versus the values for *max_size _>*1. The median values (this is true also for the mean) are always higher for these isolated orthologs than for orthologs involved in larger blocks of synteny. This confirms the results already obtained by Lemoine et al. To be exhaustive, one should note that there is a (quantitatively smaller) category of pairs that are not displayed on this plot: namely orthologs that are not involved in any clique-spine of at least 3 genomes. For all of the 4 groups, their *d_PAM _*median values are always higher than for the first group. We choose not to plot them because the co-orthology relationship may be considered as dubious in these cases. The second point to observe is that there is a clear tendency for *d_PAM _*to decrease when the synteny size increases. This effect is more pronounced for distant species (**bacteria**, **proteo **and **gamma**) than for closely related species (**entero**). In order to make sure that this effect is not related to some phylogenetic heterogeneity in the data sets, we also split the analysis by pairs of species. This is represented by the  coloured curves in Figure 5 This clearly shows that the same trend holds whatever the pair of species considered (although some species pairs have globally lower or higher values). In order to remove a possible bias arising from false positive orthologs in INPARANOID, we additionally performed the same analysis restricted to one-to-one orthologs (i.e. ignoring all groups of co-orthologs of size greater than 2 genes). The curves are then slightly shifted towards lower values of *d_PAM _*but keep the same shape as presented here. As a conclusion we can extend the results presented by Lemoine et al. in the following way: for a given pair of orthologous genes, the larger the size of the synteny in which it is involved, the more conserved the pair is.

## Conclusions

In this paper, we presented an extension of the graph alignment algorithm proposed in [23] based on a clear-cut definition of bacterial syntenies, as well as an exact algorithm to find them. The main purpose of this new extension is to allow for missing genes. More precisely, we now require genes to be present only on a quorum *q *≤ *n *of the genomes under study. Together with a very flexible definition of block of synteny (*n*-way genes aggregation in spines, presence of gap genes, partial or total conservation of the gene order), this resulted in a versatile tool to study syntenies in bacteria, that may be adapted to various kinds of studies. We presented two typical applications. The first one is related to the search of functional gene associations (for instance to the purpose of genome annotation). In this context, one should choose a quite loose gene-to-gene relationship based on sequence similarity and relaxed synteny parameters. We compared our approach to the widely used heuristics I-ADHORE. Execution times and results are very similar, showing that, at least up to ten genomes, the problem is still tractable with an exact definition and algorithm. The second application is related to evolutionary studies. In this context, it is better to work with a tighter definition of syntenies (orthology relation, clique association of genes, absence of gaps). We showed that the syntons retrieved by the algorithm presented an already documented feature: isolated pairs of orthologs are less conserved than the ones involved in larger blocks of synteny. However, we could extend this observation in two ways: first by working with multiple comparisons (i.e. by focusing on syntenies occurring on at least 3 genomes) and, second, by showing that the larger the block is, the more conserved the genes are.

Finally, we would like to stress that, although this paper and its illustrations focus on the question of syntenies, the definitions and the algorithm presented herein are applicable as well to the more general context of graphs alignments. For biological data, other possible applications could therefore concern the alignment of metabolic graphs, of *PPI *graphs or even of mixed data (e.g. metabolic versus genomic data [17]).

## Authors' contributions

Devised the algorithm: YPD AV FB MFS. Implemented the algorithm and performed the analysis: YPD AV. Wrote the paper: YPD AV MFS. All authors read and approved the final manuscript.

## Availability

The algorithm has been implemented in Java, is platform independent and is distributed as open-source (*GPL*). Source code, user's documentation and samples files are available for download at: http://www.inrialpes.fr/helix/people/viari/lxgraph

## References

[B1] NCBI: Complete Microbial Genomes http://www.ncbi.nlm.nih.gov/genomes/lproks.cgi

[B2] Huynen M Bork P Measuring genome evolution Proc Natl Acad Sci USA 1998 95 5849 5856 10.1073/pnas.95.11.5849 9600883 PMC34486

[B3] Koonin E Wolf Y Genomics of bacteria and archaea: the emerging dynamic view of the prokaryotic world Nucl Acids Res 2008 36 6688 6719 10.1093/nar/gkn668 18948295 PMC2588523

[B4] Itoh T Takemoto K Mori H Gojobori T Evolutionary Instability of Operon Structures Disclosed by Sequence Comparisons of Complete Microbial Genomes Mol Biol Evol 1999 16 332 346 10331260 10.1093/oxfordjournals.molbev.a026114

[B5] Dandekar T Snel B Huynen M P B Conservation of gene order: A fingerprint of proteins that physically interact Trends Biochem Sci 1998 23 324 328 10.1016/S0968-0004(98)01274-2 9787636

[B6] Tesler G GRIMM: genome rearrangements web server Bioinformatics 2002 18 3 492 493 10.1093/bioinformatics/18.3.492 11934753

[B7] Sinha A Meller J Cinteny: flexible analysis and visualization of synteny and genome rearrangements in multiple organisms BMC Bioinformatics 2007 8 82 10.1186/1471-2105-8-82 17343765 PMC1821339

[B8] Uno T Yagiura M Fast Algorithms to Enumerate All Common Intervals of Two Permutations Algorithmica 2000 26

[B9] Heber S Stoye J Algorithms for Finding Gene Clusters WABI: International Workshop on Algorithms in Bioinformatics, LNCS 2001

[B10] Didier G Common intervals of two sequences Algorithms in Bioinformatics Proceedings, Volume 2812 of Lecture Notes in Bioinformatics 2003 17 24

[B11] Bergeron A Corteel S Raffinot M The Algorithmic of Gene Teams WABI: International Workshop on Algorithms in Bioinformatics, Volume 2452 of Lecture Notes in Computer Science 2002 464 476

[B12] He X Goldwasser M Identifying Conserved Gene Clusters in the Presence of Homology Families J Comput Biol 2005 12 6 638 656 10.1089/cmb.2005.12.638 16108708

[B13] Pasek S Bergeron A Risler J Louis A Ollivier E Raffinot M Identification of genomic features using microsyntenies of domains: Domain teams Genome Res 2005 15 6 867 874 10.1101/gr.3638405 15899966 PMC1142477

[B14] Kim S Choi JH Yang J Gene Teams with Relaxed Proximity Constraint IEEE Computational Systems Bioinformatics Conference (CSB 2005) 2005 44 55 10.1109/csb.2005.33 16447961

[B15] Ling X He X Xin D Han J Efficiently Identifying Max-Gap Clusters in Pairwise Genome Comparison J Comput Biol 2008 15 6 593 609 10.1089/cmb.2008.0010 18631023

[B16] Ling X He X Xin D Detecting gene clusters under evolutionary constraint in a large number of genomes Bioinformatics 2009 25 5 571 577 10.1093/bioinformatics/btp027 19158161

[B17] Boyer F Morgat A Labarre L Pothier J Viari A Syntons, metabolons and interactons: an exact graph-theoretical approach for exploring neighbourhood between genomic and functional data Bioinformatics 2005 21 23 4209 4215 10.1093/bioinformatics/bti711 16216829

[B18] Calabrese P Chakravarty S Vision T Fast identification and statistical evaluation of segmental homologies in comparative maps ISMB (Supplement of Bioinformatics), Volume 19 2003 74 80 10.1093/bioinformatics/btg1008 12855440

[B19] Haas B Delcher A Wortman J Salzberg S DAGchainer: a tool for mining segmental genome duplications and synteny Bioinformatics 2004 20 18 3643 3646 10.1093/bioinformatics/bth397 15247098

[B20] Wang X Shi X Li Z Zhu Q Kong L Tang W Ge S Luo J Statistical inference of chromosomal homology based on gene colinearity and applications to Arabidopsis and rice BMC Bioinformatics 2006 7 447 10.1186/1471-2105-7-447 17038171 PMC1626491

[B21] Rödelsperger C Dieterich C Syntenator: multiple gene order alignments with a gene-specific scoring function Algorithms for Molecular Biology 2008 3 14 10.1186/1748-7188-3-14 18990215 PMC2590594

[B22] Rödelsperger C Dieterich C CYNTENATOR: progressive gene order alignment of 17 vertebrate genomes PloS one 2010 5 e8861 10.1371/journal.pone.0008861 20126624 PMC2812507

[B23] Denielou YP Boyer F Viari A Sagot MF Multiple Alignment of Biological Networks: A Flexible Approach CPM: 20th Symposium on Combinatorial Pattern Matching, Volume 5577 of Lecture Notes in Computer Science 2009 263 273

[B24] Ogata H Fujibuchi W Goto S Kanehisa M A heuristic graph comparison algorithm and its application to detect functionally related enzyme clusters Nucl Acids Res 2000 28 20 4021 4028 10.1093/nar/28.20.4021 11024183 PMC110779

[B25] Kalaev M Bafna V Sharan R Fast and Accurate Alignment of Multiple Protein Networks International Conference on Research in Computational Molecular Biology RECOMB 2008, Volume 4955 of Lecture Notes in Computer Science 2008 246 256

[B26] Simillion C Vandepoele K Saeys Y Van de Peer Y Building genomic profiles for uncovering segmental homology in the twilight zone Genome Res 2004 14 6 1095 1106 10.1101/gr.2179004 15173115 PMC419788

[B27] Remm M Storm C Sonnhammer E Automatic clustering of orthologs and in-paralogs from pairwise species comparisons J Mol Biol 2001 314 1041 1052 10.1006/jmbi.2000.5197 11743721

[B28] Altenhoff A Dessimoz C Phylogenetic and Functional Assessment of Orthologs Inference Projects and Methods PloS Comput Biol 2009 5 e1000262+ 19148271 10.1371/journal.pcbi.1000262 PMC2612752

[B29] Habib M Paul C Raffinot M Maximal Common Connected Sets of Interval Graphs CPM: 15th Symposium on Combinatorial Pattern Matching, Volume 3109 of Lecture Notes in Computer Science 2004 347 358

[B30] Vandepoele K Saeys Y Simillion C Raes J Van De Peer Y The automatic detection of homologous regions (ADHoRe) and its application to microcolinearity between Arabidopsis and rice Genome Res 2002 12 11 1792 1801 10.1101/gr.400202 12421767 PMC187543

[B31] Simillion C Janssens K Sterck L Van de Peer Y i-ADHoRe 2.0: an improved tool to detect degenerated genomic homology using genomic profiles Bioinformatics 2008 24 127 128 10.1093/bioinformatics/btm449 17947255

[B32] Cho K Lim W Math R Asraful Islam S Hong S Kim H Yun H Comparative analysis of the glg operons of Pectobacterium chrysanthemi PY35 and other prokaryotes J Mol Evol 2008 67 1 12 10.1007/s00239-008-9103-7 18594899

[B33] Montero M Almagro G Eydallin G Viale A Muñoz F Bahaji A Li J Rahimpour M Baroja-Fernàndez E Pozueta-Romero J Escherichia coli glycogen genes are organized in a single glgBXCAP transcriptional unit possessing an alternative suboperonic promoter within glgC that directs glgAP expression Biochem J 2010 433 107 117 21029047 10.1042/BJ20101186

[B34] Lin S Hanson R Cronan J Biotin synthesis begins by hijacking the fatty acid synthetic pathway Nat Chem Biol 2010 6 9 682 688 10.1038/nchembio.420 20693992 PMC2925990

[B35] Rodionov D Mironov A Gelfand M Conservation of the biotin regulon and the BirA regulatory signal in Eubacteria and Archaea Genome Res 2002 12 10 1507 1516 10.1101/gr.314502 12368242 PMC187538

[B36] Lemoine F Lespinet O Labedan B Assessing the evolutionary rate of positional orthologous genes in prokaryotes using synteny data BMC Evol Biol 2007 7 237 10.1186/1471-2148-7-237 18047665 PMC2238764

[B37] Gonnet G Hallett M Korostensky C Bernardin L Darwin v. 2:0: an interpreted computer language for the biosciences Bioinformatics 2000 16 101 103 10.1093/bioinformatics/16.2.101 10842729

